# Building a truly diverse biodiversity science

**DOI:** 10.1038/s44185-022-00003-1

**Published:** 2022-11-17

**Authors:** Joaquín Hortal, Mar Cabeza, José Alexandre F. Diniz-Filho, Sophie von der Heyden, Alycia L. Stigall, Darren C. J. Yeo

**Affiliations:** 1 Department of Biogeography and Global Change, Museo Nacional de Ciencias Naturales (MNCN-CSIC), Madrid, Spain; 2 Global Change and Conservation, Organismal and Evolutionary Biology Research Programme, Faculty of Biological and Environmental Sciences, University of Helsinki, Helsinki, Finland; 3 Departamento de Ecologia, Instituto de Ciências Biológicas—ICB, Universidade Federal de Goiás—UFG Campus II, Goiânia, GO Brazil; 4 Department of Botany and Zoology, University of Stellenbosch, Stellenbosch, South Africa; 5 Department of Earth and Planetary Sciences, University of Tennessee, Knoxville, TN USA; 6 Department of Biological Sciences, National University of Singapore, Singapore, Singapore

**Keywords:** Biodiversity

Biodiversity is an overarching concept that encompasses the variation of life at all levels of biological organization^1^. It describes all living beings, their traits and attributes, their ecological and evolutionary relationships, and all the mechanisms and processes that give rise to and maintain this vast variety of forms and functions. In this sense, biodiversity science involves both ecological and evolutionary concepts. Assuming that life on Earth had a single origin, the organismal part of biodiversity can be represented by the whole tree of life, where all species, populations and individuals that ever existed are evolutionarily interlinked, from viruses and their phages to complex animals and plants. But biodiversity is also the variety of structures, forms, colours and vigour of organic life whose increase fascinated Humboldt as he got closer to the tropics^2^. This variety further encompasses the physiological and ecosystem functions performed by each individual, which are mediated by these traits and attributes and regulated by their behaviour and life history. All of that is biodiversity. Nonetheless, the persistence of biodiversity into the future depends on how humanity interacts with natural systems, where human society can act as both custodian and consumer of natural systems. In this inaugural Editorial, we outline our views on current biodiversity science and how our journal plans to set a course toward providing a place for interdisciplinary discussion and synthesis in this necessarily diverse discipline.

## The diversity of biodiversity science

The scope of *npj Biodiversity* spans the biodiversity science continuum (https://www.nature.com/npjbiodivers/aims), encompassing a wide range of topics, from biogeography and palaeontology, to microbial and evolutionary ecology. This broad research agenda means that biodiversity scientists belong to different scientific societies, attend different meetings, and read different journals. Too often, multidisciplinary discussions are limited to coffee tables, corridors and hallways with colleagues of the same faculty, workshops on specific topics (e.g. Tropical Biology or Quaternary Evolution), synthesis centres (https://synthesis-consortium.org/) and a handful of broad-scope journals. A consequence of this fragmentation is that our perception of biodiversity pattern and process varies widely depending on which region, organism, spatial and temporal scale, ecosystem, or aspect of biodiversity is being studied. This is further complicated by the array of theoretical frameworks and analytical schools adopted by the researchers. Grasping the bigger picture of the processes behind the past and present distribution, form and functioning of life requires uniting as many of these diverse perceptions as possible.

Biodiversity research is also a science of crisis. Global change is provoking rapid environmental transformations and causing extensive declines of populations and species, often as a consequence of human activities. This is occurring through mechanisms such as overexploitation, land transformation, and human-assisted dispersal of species that later become invasive. As such, we must include social scientists and environmental economists within biodiversity research, as well as the views of native peoples and local communities. We must also recognise the conflicts between biodiversity and different models of economic development. This holistic view is vital for finding solutions that balance the needs of both biodiversity and humans.

Nevertheless, like other scientific areas, biodiversity science is affected by pervasive geographic, demographic and social biases, which create barriers and diminish the work of women, identity groups, underrepresented minorities, indigenous communities and researchers from the Global South^3–8^. These painfully consistent biases can and do have important effects on our understanding of natural processes, as these discriminated groups hold unique knowledge and viewpoints. For example, the Darwinian emphasis on the classical Malthusian view of population dynamics driven by competition for limited resources has had a tremendous, and to a considerable extent spurious, impact on our perception of ecological and evolutionary processes^9^, hampering our understanding of the widely observed coexistence of species in diverse communities^10^. Other biases include the greater attention received by the temperate regions of the Northern hemisphere^11^, compared with the attention given to drylands or other extreme habitats, or by terrestrial ecosystems compared with their marine and freshwater counterparts^12^.

## Promoting plural and thoughtful debate and synthesis in biodiversity science

*npj Biodiversity* aims to be a common forum where discoveries in all areas of biodiversity science can be discussed, so that the research in specific topics with broad implications for other disciplines permeates the whole community. This requires that scientific debates are made in egalitarian terms between people with different backgrounds and points of view. We will strive to provide safe spaces where all biodiversity research can be showcased without bias, and theoretical and practical advances can be subject to calm and civil debate. As journal editors we will implement measures to work towards a fairer and more inclusive science, such as giving proper recognition to all researchers involved in the research published^13^, or ensuring in revisions that former research made by different identity groups and local scientists is adequately acknowledged^14^. We will also acknowledge diversity by maintaining a diverse editorial board^15^ and engaging external peer-reviewers^16^ that represent local specialists, the diversity of approaches in each field, as well as early-career researchers across demographic groups. We will also encourage access to research and engage in the FAIR principles for data management and sharing^17^. Here, good practice includes making data available for reanalysis or compilation in larger databases by researchers anywhere in the world, promoting open software, and sharing reproducible code^18,19^. Our hope is that this extends the capacity of developing meta-analyses and macroecological and macroevolutionary research beyond the borders of high-income countries.

*npj Biodiversity* seeks to promote scientific discussion and synthesis. As editors, we will act as guides and moderators rather than as gatekeepers that merely decide which papers are above the threshold of publication^20^. Thus, we encourage debate as a central part of the editorial process, allowing well-grounded and clearly-identified speculation and policy-related statements in published papers when appropriate. This may include publishing non-conventional papers that foster discussion in established topics or open new research avenues^21^, if and only if they are well supported by data or published evidence. In this sense, we welcome *Comments* on areas currently under discussion, as well as *Reviews* and *Perspectives* that allow synthesis in theoretical and practical topics that are not necessarily general, but can help advance specific subdisciplines or topics. Last but not least, we want to facilitate communication between basic research and applied practitioners through *Perspectives* that translate the implications of recent research for management, conservation and adaptation to global change, or that identify which theoretical advances or additional empirical evidence would be needed to tackle specific problems.

Creating the appropriate publishing environment for journals to be true forums for debate and provide value to the scientific community is a challenging enterprise. Above all, it requires escaping from the haste imposed by the “publish or perish model”, and making an explicit effort to raise the quality of the editorial process. In *npj Biodiversity* we will seek to follow ‘slow publishing’ principles, putting emphasis on meaningful debate between authors, editors and reviewers^22^. Current research environments can prevent researchers from having time to think, but true advance stems from digesting ideas and discussing them with the detail, depth and time they may need (http://slow-science.org/)^23–25^. Therefore, to contribute to a healthier, gentler and more thoughtful approach to biodiversity science, we will provide thorough and thoughtful reviews. We will make editorial decisions that, when paired with equally thorough and thoughtful work by authors, can reduce the number of times a paper bounces back and forth in successive rounds of peer review and revision. Note that this does not necessarily mean longer editorial times! Paradoxically, when authors, reviewers and editors commit to these “slow” publishing principles, the publication process can speed up. And most importantly, it will promote the spirit of productive debate that we aim for in *npj Biodiversity*.

## Concluding remarks

Contemporary biodiversity science is diverse, but also fragmented and plagued by biases. Ameliorating these issues is the responsibility of our research community, of which we as Editors are part. We aim to create an environment for truly diverse access to global biodiversity science, where all regions and demographic groups contribute to a better understanding of the living world. *npj Biodiversity* will do this by showcasing high-quality ecological and evolutionary research, fostering debate, and promoting a truly diverse, open and welcoming biodiversity research community.

## References

[1] Gaston, K. J. & Spicer, J. I. *Biodiversity: An Introduction*. Second edition. (Blackwell, 2004).

[2] Humboldt, A. V. *Ansichten der Natur, mit wissenschaftlichen Erläuterungen* (J. G. Cotta, 1808).

[3] Mott, C. & Cockayne, D. Citation matters: mobilizing the politics of citation toward a practice of ‘conscientious engagement’. *Gender, Place Culture* **24**, 954–973 (2017). 10.1080/0966369X.2017.1339022

[4] Smith, C. A. & Garrett-Scott, D. “We are not named”: black women and the politics of citation in anthropology. *Feminist Anthropol.* **2**, 18–37 (2021). 10.1002/fea2.12038

[5] Grosso, J. et al. Male homophily in South American herpetology: one of the major processes underlying the gender gap in publications. *Amphibia-Reptilia* **42**, 407–418 (2021).

[6] Monarrez, P. M. et al. Our past creates our present: a brief overview of racism and colonialism in Western paleontology. *Paleobiology* **48**, 173–185 (2022). 10.1017/pab.2021.28

[7] Martínez-Blancas, A. et al. Surviving racism and sexism in academia: Sharing experiences, insights, and perspectives. *Bull. Ecol. Soc. Am.* e2033 10.1002/bes2.2033 (2022).

[8] von der Heyden, S. Environmental DNA surveys of African biodiversity: state of knowledge, challenges, and opportunities. *Environmental DNA*10.1002/edn3.363 (2022).

[9] Muñoz-Rubio, J. Competition as a dominant concept in ecology: on the unity of science and ideology. *Ludus Vitalis* **11**, 3–24 (2003).

[10] Simha, A., Pardo-De la Hoz, C. J. & Carley, L. N. Moving beyond the “Diversity Paradox”: the limitations of competition-based frameworks in understanding species diversity. *Am. Naturalist* **200**, 89–100 (2022). 35737981 10.1086/720002

[11] Eichhorn, M. P., Baker, K. & Griffiths, M. Steps towards decolonising biogeography. *Front. Biogeography* e44795 10.21425/F5FBG44795 (2020).

[12] Di Marco, M. et al. Changing trends and persisting biases in three decades of conservation science. *Global Ecol. Conser.* **10**, 32–42 (2017). 10.1016/j.gecco.2017.01.008

[13] Allen, L., Scott, J., Brand, A., Hlava, M. & Altman, M. Credit where credit is due. *Nature* **508**, 312–313 (2014). 24745070 10.1038/508312a

[14] Gorneau, J. A. et al. Framing the future for taxonomic monography: Improving recognition, support, and access. *Bull. Soc. Systematic Biologists* **1**, 8328 (2022). 10.18061/bssb.v1i1.8328

[15] Grogan, K. E. How the entire scientific community can confront gender bias in the workplace. *Nat. Ecol. Evol.* **3**, 3–6 (2019). 30478306 10.1038/s41559-018-0747-4

[16] Murray, D. et al. Author-reviewer homophily in peer review. Preprint at *bioRxiv*10.1101/400515 (2019).

[17] Wilkinson, M. D. et al. The FAIR guiding principles for scientific data management and stewardship. *Sci. Data* **3**, 160018 (2016). 26978244 10.1038/sdata.2016.18 PMC4792175

[18] Cooper, N. & Hsing, P.-Y. A guide to reproducible code in ecology and evolution. *Br. Ecol. Soc. Lond.* https://www.britishecologicalsociety.org/wp-content/uploads/2017/12/guide-to-reproducible-code.pdf (2017).

[19] Grayson, K. L., Hilliker, A. K. & Wares, J. R. R Markdown as a dynamic interface for teaching: modules from math and biology classrooms. *Mathematical Biosci.* **349**, 108844 (2022). 10.1016/j.mbs.2022.108844 PMC9487201 35623397

[20] Dawson, M. N., Field, R. & Hortal, J. Guides, not gatekeepers. *Front. Biogeography* **6**, 108–110 (2014). 10.21425/F56324109

[21] Riera, R. & Rodríguez, R. What if peer-review process is killing thinking-out-of-the-box science? *Front. Marine Sc.* **9**, 924469 (2022). 10.3389/fmars.2022.924469

[22] Hortal, J., Meyer, C., Bourguet, D. & Dawson, M. N. Slow publishing in the age of ‘fast-food’. *Front. Biogeography* **11**, e44213 (2019). 10.21425/F5FBG44213

[23] Alleva, L. Taking time to savour the rewards of slow science. *Nature* **443**, 271 (2006). 16988684 10.1038/443271e

[24] Halme, P., Komonen, A. & Huitu, O. Solutions to replace quantity with quality in science. *Trends Ecol. Evol.* **27**, 586 (2012). 22959279 10.1016/j.tree.2012.08.007

[25] Lutz, J. F. Slow science. *Nat. Chemistry* **4**, 588–589 (2012). 10.1038/nchem.1415 22824878

